# 3-*O*-Ethyl-l-ascorbic acid

**DOI:** 10.1107/S1600536808009963

**Published:** 2008-04-16

**Authors:** Shu Jin, Xiaoqin Miao

**Affiliations:** a Nanjing Research Institute for Comprehensive Utilization of Wild Plants, Jiangwangmiaojie 4#, Nanjing 210042, People’s Republic of China

## Abstract

In the crystal structure of the title compound, C_8_H_12_O_6_, mol­ecules are linked to each other by O—H⋯O hydrogen bonding.

## Related literature

For general background, see: Nihro *et al.* (1992 ▶); Satoh *et al.* (1994 ▶).
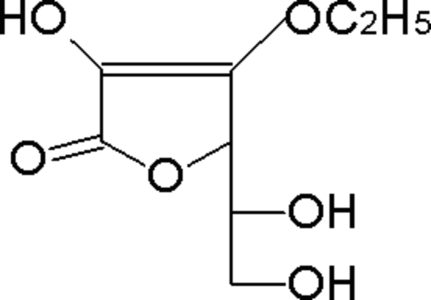


## Experimental

### 

#### Crystal data

C_8_H_12_O_6_
*M* *_r_* = 204.18
Orthorhombic, 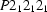

*a* = 4.6690 (9) Å
*b* = 11.939 (2) Å
*c* = 16.794 (3) Å
*V* = 936.2 (3) Å^3^
*Z* = 4
Mo *K*α radiation
μ = 0.13 mm^−1^
*T* = 293 (2) K
0.20 × 0.20 × 0.10 mm

#### Data collection

Enraf–Nonius CAD-4 diffractometer
Absorption correction: none
1973 measured reflections
1024 independent reflections
882 reflections with *I* > 2σ(*I*)
*R*_int_ = 0.028
3 standard reflections every 200 reflections intensity decay: none

#### Refinement

*R*[*F* ^2^ > 2σ(*F* ^2^)] = 0.040
*wR*(*F* ^2^) = 0.138
*S* = 1.00
1024 reflections
137 parameters
1 restraint
H atoms treated by a mixture of independent and constrained refinement
Δρ_max_ = 0.18 e Å^−3^
Δρ_min_ = −0.26 e Å^−3^

### 

Data collection: *CAD-4 Software* (Enraf–Nonius, 1989 ▶); cell refinement: *CAD-4 Software*; data reduction: *XCAD4* (Harms & Wocadlo, 1995 ▶); program(s) used to solve structure: *SHELXTL* (Sheldrick, 2008 ▶); program(s) used to refine structure: *SHELXTL*; molecular graphics: *SHELXTL*; software used to prepare material for publication: *SHELXTL*.

## Supplementary Material

Crystal structure: contains datablocks I, global. DOI: 10.1107/S1600536808009963/xu2411sup1.cif

Structure factors: contains datablocks I. DOI: 10.1107/S1600536808009963/xu2411Isup2.hkl

Additional supplementary materials:  crystallographic information; 3D view; checkCIF report

## Figures and Tables

**Table 1:** Hydrogen-bond geometry (Å, °)

*D*—H⋯*A*	*D*—H	H⋯*A*	*D*⋯*A*	*D*—H⋯*A*
O2—H2*A*⋯O5^i^	0.83 (3)	2.06 (3)	2.873 (3)	168 (5)
O5—H5*A*⋯O3^ii^	0.91 (5)	1.90 (4)	2.748 (4)	154 (4)
O6—H6*A*⋯O6^iii^	0.87 (5)	1.87 (5)	2.715 (4)	163 (4)

Symmetry codes: (i) 
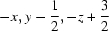
; (ii) 
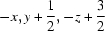
; (iii) 
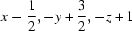
.

**Fig. 1.**
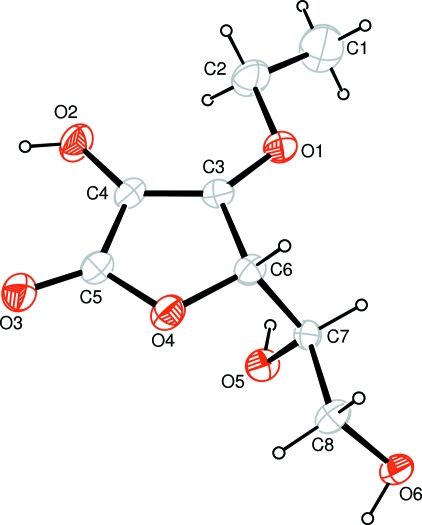
The molecular structure of (I), with atom labels and 30% probability displacement ellipsoids for non-H atoms.

## supplementary crystallographic information

### Comment

L-Ascorbic acid has been widely employed as an antioxidant for stabilization of nutrients. However, the low lipophilicity of it and its susceptibility to thermal and oxidative degradation restricts its field of application and has raised considerable interest in the study of ascorbic acid derivatives with increased lipophilicity and stability. The title compound is one of the lipophilic ascorbic acid derivatives, which exhibit antioxidative properties (Nihro *et al.*,1992) and can be used as antioxidant in food (Satoh *et al.*, 1994). As part of our ongoing study on ascorbic acid derivatives, we report here the crystal structure of the title compound (Fig. 1).

The geometrical parameters of the compound are normal. The C3—C4 bond distance of 1.332 (5) Å and O3—C5 bond distance of 1.215 (4) Å indicate typical C═C and O═C double bonds. Molecules are linked to each other by O—H···O hydrogen bonding (Table 1).

### Experimental

0.1 mol 5,6-*O*,*O*-Isopropylidene L-ascorbic acid was dissolved in 100 ml DMSO at room temperature, and 0.12 mol NaHCO_3_ was added with stirring. After the addition of 0.1 mol ethyl bromide, the mixture was stirred at 313 K for 6 h. The solvent was distilled of at 333 K under reduced pressure. The residue was dissolved in 50 ml water and extracted five times with ethyl acetate (100 ml/time). The collected organic phase was dried over Na_2_SO_4_ and the solvent was evaporated at reduced pressure. 100 ml 0.1 *M* HCl was added to the residue, refluxed for 15 min and then the solvent was evaporated at reduced pressure. The residue was dissolved in ethyl acetate; single crystals were obtained by slow evaporation of the ethyl acetate solution.

### Refinement

Hydroxyl H atoms were located in a difference Fourier map and positional parameters were refined, *U*_iso_(H) = 1.5*U*_eq_(O). Other H atoms were positioned geometrically with C—H = 0.96–0.98 Å and refined using a riding model with *U*_iso_(H) = 1.2*U*_eq_(C) or 1.5*U*_eq_(C) (for methyl). As no significant anomalous scattering effect, Friedel pairs were merged.

### Crystal data

**Table:**

C_8_H_12_O_6_	*D*_x_ = 1.449 Mg m^−^^3^
*M**_r_* = 204.18	Melting point: 385 K
Orthorhombic, *P*2_1_2_1_2_1_	Mo *K*α radiation λ = 0.71073 Å
Hall symbol: P 2ac 2ab	Cell parameters from 25 reflections
*a* = 4.6690 (9) Å	θ = 10–13º
*b* = 11.939 (2) Å	µ = 0.13 mm^−^^1^
*c* = 16.794 (3) Å	*T* = 293 (2) K
*V* = 936.2 (3) Å^3^	Plate, colourless
*Z* = 4	0.20 × 0.20 × 0.10 mm
*F*_000_ = 432	

### Data collection

**Table:**

Enraf–Nonius CAD-4 diffractometer	*R*_int_ = 0.028
Radiation source: fine-focus sealed tube	θ_max_ = 25.2º
Monochromator: graphite	θ_min_ = 2.1º
*T* = 293(2) K	*h* = 0→5
ω/2θ scans	*k* = 0→14
Absorption correction: none	*l* = −20→20
1973 measured reflections	3 standard reflections
1024 independent reflections	every 200 reflections
882 reflections with *I* > 2σ(*I*)	intensity decay: none

### Refinement

**Table:**

Refinement on *F*^2^	Secondary atom site location: difference Fourier map
Least-squares matrix: full	Hydrogen site location: inferred from neighbouring sites
*R*[*F*^2^ > 2σ(*F*^2^)] = 0.040	H atoms treated by a mixture of independent and constrained refinement
*wR*(*F*^2^) = 0.138	*w* = 1/[σ^2^(*F*_o_^2^) + (0.1*P*)^2^ + 1.3*P*] where *P* = (*F*_o_^2^ + 2*F*_c_^2^)/3
*S* = 1.00	(Δ/σ)_max_ < 0.001
1024 reflections	Δρ_max_ = 0.18 e Å^−^^3^
137 parameters	Δρ_min_ = −0.26 e Å^−^^3^
1 restraint	Extinction correction: SHELXL, Fc^*^=kFc[1+0.001xFc^2^λ^3^/sin(2θ)]^-1/4^
Primary atom site location: structure-invariant direct methods	Extinction coefficient: 0.121 (14)

### Special details

**Table:**

Experimental. ^1^H NMR (500 MHz, CDCl3): δ1.39 (3H, t), 3.86 (2H, m), 3.96 (1H, m), 4.54 (2H, q), 4.71 (1H, d) p.p.m.
Geometry. All e.s.d.'s (except the e.s.d. in the dihedral angle between two l.s. planes) are estimated using the full covariance matrix. The cell e.s.d.'s are taken into account individually in the estimation of e.s.d.'s in distances, angles and torsion angles; correlations between e.s.d.'s in cell parameters are only used when they are defined by crystal symmetry. An approximate (isotropic) treatment of cell e.s.d.'s is used for estimating e.s.d.'s involving l.s. planes.
Refinement. Refinement of *F*^2^ against ALL reflections. The weighted *R*-factor *wR* and goodness of fit *S* are based on *F*^2^, conventional *R*-factors *R* are based on *F*, with *F* set to zero for negative *F*^2^. The threshold expression of *F*^2^ > σ(*F*^2^) is used only for calculating *R*-factors(gt) *etc*. and is not relevant to the choice of reflections for refinement. *R*-factors based on *F*^2^ are statistically about twice as large as those based on *F*, and *R*- factors based on ALL data will be even larger.

### Fractional atomic coordinates and isotropic or equivalent isotropic displacement parameters (Å^2^)

**Table:**

	*x*	*y*	*z*	*U*_iso_*/*U*_eq_	
O1	0.5474 (6)	0.5579 (2)	0.82156 (13)	0.0513 (7)	
C1	0.6281 (17)	0.6421 (5)	0.9472 (3)	0.0896 (18)	
H1A	0.5996	0.6352	1.0036	0.134*	
H1B	0.5371	0.7093	0.9285	0.134*	
H1C	0.8295	0.6455	0.9359	0.134*	
O2	0.1035 (7)	0.3667 (2)	0.85756 (15)	0.0563 (8)	
H2A	0.027 (12)	0.305 (2)	0.851 (3)	0.084*	
C2	0.5046 (14)	0.5463 (4)	0.90741 (19)	0.0664 (14)	
H2B	0.3016	0.5418	0.9192	0.080*	
H2C	0.5953	0.4782	0.9262	0.080*	
C3	0.4076 (7)	0.4855 (2)	0.77537 (18)	0.0385 (8)	
O3	−0.0350 (7)	0.2854 (2)	0.69573 (15)	0.0555 (7)	
C4	0.2202 (8)	0.4033 (3)	0.78834 (19)	0.0401 (8)	
O4	0.2730 (6)	0.41543 (19)	0.65249 (13)	0.0451 (7)	
C5	0.1347 (8)	0.3595 (3)	0.7110 (2)	0.0419 (8)	
O5	0.0863 (5)	0.64292 (19)	0.67658 (15)	0.0419 (6)	
H5A	0.088 (11)	0.671 (3)	0.727 (3)	0.063*	
O6	0.3693 (6)	0.7318 (2)	0.53918 (14)	0.0466 (7)	
H6A	0.213 (11)	0.729 (4)	0.511 (3)	0.070*	
C6	0.4518 (7)	0.4996 (2)	0.68745 (17)	0.0355 (8)	
H6B	0.6523	0.4838	0.6744	0.043*	
C7	0.3734 (7)	0.6151 (3)	0.65672 (17)	0.0329 (7)	
H7A	0.5026	0.6705	0.6804	0.039*	
C8	0.4004 (9)	0.6198 (3)	0.56722 (18)	0.0427 (9)	
H8A	0.5861	0.5910	0.5514	0.051*	
H8B	0.2544	0.5729	0.5432	0.051*	

### Atomic displacement parameters (Å^2^)

**Table:**

	*U*^11^	*U*^22^	*U*^33^	*U*^12^	*U*^13^	*U*^23^
O1	0.0599 (17)	0.0583 (14)	0.0356 (11)	−0.0134 (14)	−0.0067 (13)	0.0078 (11)
C1	0.122 (5)	0.091 (3)	0.056 (3)	−0.020 (4)	0.007 (3)	−0.013 (2)
O2	0.0731 (19)	0.0566 (14)	0.0391 (12)	−0.0154 (17)	0.0083 (14)	0.0088 (12)
C2	0.096 (4)	0.068 (2)	0.0350 (16)	−0.016 (3)	−0.005 (2)	0.0046 (17)
C3	0.0394 (17)	0.0395 (15)	0.0365 (15)	0.0018 (17)	−0.0037 (15)	0.0049 (13)
O3	0.0678 (17)	0.0441 (13)	0.0544 (14)	−0.0087 (15)	−0.0043 (15)	0.0031 (11)
C4	0.0452 (19)	0.0366 (16)	0.0385 (16)	0.0016 (15)	0.0020 (16)	0.0092 (13)
O4	0.0605 (16)	0.0383 (12)	0.0364 (11)	−0.0028 (12)	0.0032 (12)	0.0020 (9)
C5	0.0473 (19)	0.0352 (15)	0.0432 (18)	0.0034 (18)	0.0011 (17)	0.0056 (14)
O5	0.0367 (13)	0.0485 (12)	0.0406 (12)	0.0064 (12)	0.0060 (11)	0.0020 (10)
O6	0.0455 (14)	0.0504 (13)	0.0440 (13)	−0.0009 (13)	−0.0044 (12)	0.0163 (11)
C6	0.0328 (16)	0.0358 (15)	0.0378 (15)	0.0055 (15)	0.0032 (14)	0.0039 (13)
C7	0.0293 (15)	0.0367 (15)	0.0326 (15)	−0.0029 (14)	0.0023 (13)	0.0023 (12)
C8	0.054 (2)	0.0394 (16)	0.0345 (16)	0.0045 (19)	0.0078 (17)	0.0044 (14)

### Geometric parameters (Å, °)

**Table:**

O1—C3	1.332 (4)	C4—C5	1.456 (5)
O1—C2	1.462 (4)	O4—C5	1.353 (4)
C1—C2	1.444 (7)	O4—C6	1.432 (4)
C1—H1A	0.9600	O5—C7	1.421 (4)
C1—H1B	0.9600	O5—H5A	0.91 (4)
C1—H1C	0.9600	O6—C8	1.425 (4)
O2—C4	1.356 (4)	O6—H6A	0.87 (5)
O2—H2A	0.83 (3)	C6—C7	1.516 (4)
C2—H2B	0.9700	C6—H6B	0.9800
C2—H2C	0.9700	C7—C8	1.510 (4)
C3—C4	1.332 (5)	C7—H7A	0.9800
C3—C6	1.500 (4)	C8—H8A	0.9700
O3—C5	1.215 (4)	C8—H8B	0.9700
			
C3—O1—C2	116.5 (3)	O3—C5—C4	129.0 (3)
C2—C1—H1A	109.5	O4—C5—C4	109.9 (3)
C2—C1—H1B	109.5	C7—O5—H5A	107 (3)
H1A—C1—H1B	109.5	C8—O6—H6A	103 (4)
C2—C1—H1C	109.5	O4—C6—C3	104.2 (2)
H1A—C1—H1C	109.5	O4—C6—C7	111.0 (3)
H1B—C1—H1C	109.5	C3—C6—C7	113.8 (3)
C4—O2—H2A	110 (4)	O4—C6—H6B	109.2
C1—C2—O1	109.1 (4)	C3—C6—H6B	109.2
C1—C2—H2B	109.9	C7—C6—H6B	109.2
O1—C2—H2B	109.9	O5—C7—C8	107.7 (3)
C1—C2—H2C	109.9	O5—C7—C6	111.1 (3)
O1—C2—H2C	109.9	C8—C7—C6	110.7 (3)
H2B—C2—H2C	108.3	O5—C7—H7A	109.1
O1—C3—C4	134.8 (3)	C8—C7—H7A	109.1
O1—C3—C6	115.7 (3)	C6—C7—H7A	109.1
C4—C3—C6	109.5 (3)	O6—C8—C7	110.8 (3)
C3—C4—O2	129.9 (3)	O6—C8—H8A	109.5
C3—C4—C5	107.4 (3)	C7—C8—H8A	109.5
O2—C4—C5	122.6 (3)	O6—C8—H8B	109.5
C5—O4—C6	109.1 (2)	C7—C8—H8B	109.5
O3—C5—O4	121.1 (3)	H8A—C8—H8B	108.1
			
C3—O1—C2—C1	−169.9 (4)	C5—O4—C6—C3	1.1 (3)
C2—O1—C3—C4	3.1 (6)	C5—O4—C6—C7	−121.7 (3)
C2—O1—C3—C6	179.3 (3)	O1—C3—C6—O4	−178.1 (3)
O1—C3—C4—O2	1.1 (6)	C4—C3—C6—O4	−0.9 (4)
C6—C3—C4—O2	−175.3 (3)	O1—C3—C6—C7	−57.1 (4)
O1—C3—C4—C5	176.8 (4)	C4—C3—C6—C7	120.0 (3)
C6—C3—C4—C5	0.4 (4)	O4—C6—C7—O5	61.3 (3)
C6—O4—C5—O3	178.3 (3)	C3—C6—C7—O5	−55.8 (4)
C6—O4—C5—C4	−1.0 (4)	O4—C6—C7—C8	−58.3 (4)
C3—C4—C5—O3	−178.9 (4)	C3—C6—C7—C8	−175.4 (3)
O2—C4—C5—O3	−2.8 (6)	O5—C7—C8—O6	67.1 (4)
C3—C4—C5—O4	0.3 (4)	C6—C7—C8—O6	−171.2 (3)
O2—C4—C5—O4	176.4 (3)		

### Hydrogen-bond geometry (Å, °)

**Table:**

*D*—H···*A*	*D*—H	H···*A*	*D*···*A*	*D*—H···*A*
O2—H2A···O5^i^	0.83 (3)	2.06 (3)	2.873 (3)	168 (5)
O5—H5A···O3^ii^	0.91 (5)	1.90 (4)	2.748 (4)	154 (4)
O6—H6A···O6^iii^	0.87 (5)	1.87 (5)	2.715 (4)	163 (4)

Symmetry codes: (i) −*x*, *y*−1/2, −*z*+3/2; (ii) −*x*, *y*+1/2, −*z*+3/2; (iii) *x*−1/2, −*y*+3/2, −*z*+1.
